# Genotypic Variation of Endophytic Nitrogen-Fixing Activity and Bacterial Flora in Rice Stem Based on Sugar Content

**DOI:** 10.3389/fpls.2021.719259

**Published:** 2021-08-10

**Authors:** Takanori Okamoto, Rina Shinjo, Arisa Nishihara, Kazuma Uesaka, Aiko Tanaka, Daisuke Sugiura, Motohiko Kondo

**Affiliations:** ^1^Graduate School of Bioagricultural Sciences, Nagoya University, Nagoya, Japan; ^2^Bioproduction Research Institute, National Institute of Advanced Industrial Science and Technology, Tsukuba, Japan; ^3^Center for Gene Research, Nagoya University, Nagoya, Japan

**Keywords:** bacterial flora, endophyte, *nifH*, nitrogen fixation, non-structural carbohydrate, rice, stem

## Abstract

Enhancement of the nitrogen-fixing ability of endophytic bacteria in rice is expected to result in improved nitrogen use under low-nitrogen conditions. Endophytic nitrogen-fixing bacteria require a large amount of energy to fix atmospheric nitrogen. However, it is unknown which carbon source and bacteria would affect nitrogen-fixing activity in rice. Therefore, this study examined genotypic variations in the nitrogen-fixing ability of rice plant stem as affected by non-structural carbohydrates and endophytic bacterial flora in field-grown rice. In the field experiments, six varieties and 10 genotypes of rice were grown in 2017 and 2018 to compare the acetylene reduction activity (nitrogen-fixing activity) and non-structural carbohydrates (glucose, sucrose, and starch) concentration in their stems at the heading stage. For the bacterial flora analysis, two genes were amplified using a primer set of 16S rRNA and nitrogenase (NifH) gene-specific primers. Next, acetylene reduction activity was correlated with sugar concentration among genotypes in both years, suggesting that the levels of soluble sugars influenced stem nitrogen-fixing activity. Bacterial flora analysis also suggested the presence of common and genotype-specific bacterial flora in both 16S rRNA and *nifH* genes. Similarly, bacteria classified as rhizobia, such as *Bradyrhizobium* sp. (*Alphaproteobacteria*) and *Paraburkholderia* sp. (*Betaproteobacteria*), were highly abundant in all rice genotypes, suggesting that these bacteria make major contributions to the nitrogen fixation process in rice stems. *Gammaproteobacteria* were more abundant in CG14 as well, which showed the highest acetylene reduction activity and sugar concentration among genotypes and is also proposed to contribute to the higher amount of nitrogen-fixing activity.

## Introduction

Cultivated rice, *Oryza sativa* L., which originates from Asia, is now widely propagated. This rice species feeds about half of the population of the world (Sasaki, 2008). Another cultivated rice species, *O. glaberrima* Steud., has been domesticated in West African countries, and continues to be cultivated, although its production is decreasing (Futakuchi et al., 2012). Nitrogen (N) availability limits rice yield since N is an essential element for plants to sustain photosynthesis and other important functions by decomposing proteins, nucleic acids, and other important organic compounds. Therefore, to meet the food demands of the growing population since the industrial revolution, the use of chemical N fertilizers produced through the Haber–Bosch process has enabled rapid growth of food production, including rice (Smil, 2002). However, the production of chemical N fertilizers requires fossil energy, and the excessive use of N fertilizers causes serious environmental problems, worldwide (Good and Beatty, 2011). Alternatively, due to its cost, N fertilizers are inadequately supplied to many developing and rapidly growing countries. This results in a situation where N availability critically limits the biomass production and yields of crops, including rice (Vitousek et al., 2009; Rogers and Oldroyd, 2014).

The efficient use of biological nitrogen fixation (BNF) is expected to function as an alternative to Haber–Bosch process. In this alternative process catalyzed by the nitrogenase enzyme in bacteria and archaea, atmospheric N_2_ is reduced to ammonia (Dixon and Kahn, 2004). Rice paddy systems have a high N-fixing ability and can maintain N fertility due to various forms of BNF, including the ones free living in the soil, associative in the rhizosphere, or endophytic in plants (Yoshida and Ancajas, 1973; Pittol et al., 2016). Therefore, the enhancement of these BNF capabilities during rice production would solve the problems of the over- and under-use of N fertilizers.

Furthermore, among BNF types, leguminous crops forming rhizobial symbiosis in nodules have been of the most interest as N-fixing agents in agricultural systems (Herridge et al., 2008). Besides leguminous rhizobia symbiosis, significant contribution to BNFs by endophytes is known in other crops, including rice (James and Olivares, 1998). Petrini (1991) has therefore defined endophytes as organisms inhabiting plant organs that, at some time in their life, can colonize an internal plant tissue without causing harm to their host. Sugarcane in Brazil (Lima et al., 1987), sweet potato in Japan (Yoneyama et al., 1998), and pineapples in Thailand (Ando et al., 2000) were reported to possess high BNF capacities by endophytes, thereby contributing significantly to plant-N nutrition. In rice plants as well, the root favors N-fixing bacteria partly due to its anaerobic survival environment under submerged conditions (Yoshida and Ancajas, 1973). Additionally in the root, the lower part of the stem, including the leaf sheath and culm, is an active N-fixing site (Ito et al., 1980). Significant genotypic differences in the contribution of endophytic BNF in whole rice plants to total N accumulation in the range of 1.5–21% have also been observed (Shrestha and Ladha, 1996). Therefore, it would be valuable to determine the factors responsible for these genotypic differences during endophytic N fixation and identify the major bacteria inhabiting the stem.

Since BNF by nitrogenase requires a large amount of energy, carbon supply from the host plant is one of the important factors to enhance the contribution of endophytic N fixation. Thus, in the case of nodules in legumes, the host plant provides carbon sources for bacteroides in the form of dicarboxylates, particularly malate and succinate (Day and Copeland, 1991), while bacteroides provide fixed N for the host plant in the form of amides or ureids or both. Therefore, the major habitat of endophytes is the intercellular spaces that contain non-structural carbohydrates (NSCs), amino acids, and inorganic nutrients, which support the growth of endophytes (Bacon and Hinton, 2006). Among non-leguminous plants, those with high sugar contents, such as sugarcane, sweet potato, and pineapple have significant endophytic N-fixing activity as mentioned above.

Similarly, in rice, the stem has an important role as the storage organ for NSC. The NSC is stored in the culms and leaf sheaths until heading or anthesis and contributes to the part of grain yield through the translocation of NSC to the panicle during grain filling (Yoshida, 1972). Large variations exist in the accumulation and components of NSCs among rice varieties (Arai-Sanoh et al., 2011). *Oryza sativa* was also reported to accumulate higher starch than *O. glaberrima* (Sakagami et al., 1999). High-yielding indica varieties accumulate more NSCs in the stem than standard japonica varieties at the heading stage (Yoshinaga et al., 2013). Recently, the varieties for animal feeds that accumulate high NSC, sugar, and starch have also been developed to improve its nutritional value as whole-crop silage (Ookawa et al., 2010; Matsushita et al., 2011, 2014). Several mutant lines differing in the genes, which are involved in NSC accumulation have also been developed by *Tos17* (Miyao et al., 2003). Specifically, the *agpl1*, which lacks a starch synthesis gene *OsAGPL1*, showed lower starch in the stems; and the *lse1*, which lacks a starch degradation gene, *OsGWD1*, showed a 10-fold higher starch content in leaf blades compared to the wild type cv. Nipponbare (Hirose et al., 2006, 2013; Okamura et al., 2013). Another factor affecting NSC levels in stems is environmental conditions, especially light conditions. From previous studies, the shading treatment from the elongation to heading stage reduced the levels of starch and sucrose in rice stems (Wu et al., 2017), which might affect N-fixing activity as well. However, the effect of the variations in NSC accumulation on N fixation in the stem has not been investigated.

Recently, there has been a tremendous progress in understanding N-fixing endophytic microflora in non-leguminous crops, including rice. On the basis of the studies conducted, active expression of NifH, one of the proteins that compose the nitrogenase enzyme, is phylogenetically similar to that of the *Bradyrhizobium* sp., and *Azorhizobium* sp. was observed in the N-fixing sugarcane stems, sweet potato stems, and tuberous roots (Terakado-Tonooka et al., 2008; Fischer et al., 2012; Yoneyama et al., 2019). In rice roots as well, active BNF by methane-oxidizing methanotrophs on consuming CH_4_ as a carbon source has also been reported (Bao et al., 2014; Shinoda et al., 2019). The presence of N-fixing genes (*nifHDK*) has been confirmed in rice shoots as well (Okubo et al., 2014). Furthermore, observations by fluorescence and electron microscopy have demonstrated that the N-fixing bacteria, the *Herbaspirillum* sp. strain B501, colonizes the intercellular shoot spaces in wild rice (Elbeltagy et al., 2001). However, N-fixing microflora in the rice shoot, especially in the lower part of the stem where N-fixing activity is particularly high, and their variations among rice varieties are largely unknown since there has been no analysis of the bacterial flora targeting the nitrogenase genes.

Therefore, this study examined the genotypic differences in N-fixing activities by measuring these acetylene reduction activities (ARAs) and the impact of NSC and N concentration in stems have on ARA using several rice varieties, including *O. sativa* and *O. glaberrima*. Mutant lines that differ in NSC accumulation in the stem, such as *agpl1* and *lse1* were also used. Additionally, the effect of the change in NSC concentration created by shading treatment on N-fixing ability was investigated. We also estimated major N-fixing bacterial species that contribute to N fixation in the stem and their genotypic variations using the 16S rRNA and nitrogenase (NifH) genes in amplicon sequencing. Possible relationships between major N-fixing bacterial species and carbon sources were discussed as well.

## Materials and Methods

### Field Experiments

Field experiments were conducted in 2017 and 2018 at the Togo Field Science and Education Center (Togo field) of Nagoya University, Aichi, Japan (35°6′36N, 137°4′59E). The contents of C and N in the soil were found to be 1.99 and 0.19%, respectively. Five varieties of *O. sativa* (Nipponbare, Leafstar, Tachisuzuka, Tachiayaka, and Hokuriku 193), including a variety of *O. glaberrima* (CG14) were used in 2017. However, six varieties of *O. sativa* L. (Nipponbare, Leafstar, Tachisuzuka, Hokuriku 193, Takanari, and Habataki), two varieties of *O. glaberrima* (CG14 and WK18), and two mutant lines of Nipponbare (*agpl1* and *lse1*) were used in 2018 (Table 1). Nipponbare, which was the first whole-genome sequenced in cereal crops (Matsumoto et al., 2005), was used as a standard variety in this study.

**Table 1:** Rice varieties and lines used at field and pot experiments.

**Rice genotypes**	**Species**	**Subgroup**	**Characteristics**
Nipponbare	*O. sativa*	*Japonica*	Standard japonica variety
*agpl1*	*O. sativa*	*Japonica*	High sugar accumulation in the shoot (Homozygous *OsAGPL1* mutant lines of Nipponbare)
*lse1*	*O. sativa*	*Japonica*	High starch accumulation in the leaf (Leaf Starch Excess 1 mutant line of Nipponbare)
Leafstar	*O. sativa*	*Japonica-Indica*	Forage use
Tachisuzuka	*O. sativa*	*Japonica-Indica*	Forage use
Tachiayaka	*O. sativa*	*Japonica-Indica*	Forage use
Hokuriku193	*O. sativa*	*Indica*	High-yielding
Takanari	*O. sativa*	*Indica*	High-yielding semi-dwarf
Habataki	*O. sativa*	*Indica*	High-yielding semi-dwarf
CG14	*O. glaberrima*	-	Landrace from Senegal, Africa
WK18	*O. glaberrima*	-	Landrace from Mali, Africa

Sowing and transplanting were conducted on May 8 and May 29 in 2017, and May 8 and May 30 in 2018, respectively. Then, the seedling was transplanted at a density of 22.2 hills m^−2^ (15 × 30 cm), with one plant per hill. The plot size of each rice genotype was 1.5-m wide × 3.0-m long with one replicated plot in 2017 and 1.5-m wide × 1.5-m long with two replicated plots in 2018. As a basal dressing, chemical fertilizers were also applied at the rates of 3 g P_2_O_5_ m^−2^ and 3 g K_2_O m^−2^, whereas 6 g N m^−2^ was used as a controlled release fertilizer (LP40: LPS100: LP140 = 1:1:1, JCAM AGRI Co. Ltd. Tokyo, Japan). LP40, LPS100, and LP140 fertilizers release 80% of their total N content at 40, 100, and at 140 days after application at 25°C, respectively. Plants were grown under conventional cultural practices. Furthermore, the soil was kept flooded to a depth of about 5 cm from transplanting to harvest, except during the one-week-long midseason drainage, which started at 46 day after transplanting (DAT) in 2017 and 48 DAT in 2018.

The sampling was conducted at the heading stage, because the stem biomass generally reaches its maximum and the ARA per plant also showed the highest value at this stage (Ladha et al., 1987). Three plants in 2017 and four plants, two plants from each of the two replicated plots, in 2018 were sampled at the heading stage (0–50% of tillers showed panicle emergence) in all genotypes for evaluating N-fixing activity by ARA (Burris, 1972), NSC, N content in both 2017 and 2018, and DNA extraction in 2018. The main stem and other three stems with similar thickness in each plant were sampled and cut into 10 cm lengths from the stem base; however, only Nipponbare was sampled at the four growth stages in 2018. The growth stages of Nipponbare for plant sampling were at the tillering stage on 45 DAT, panicle initiation stage on 64 DAT, heading stage on 79 DAT (same sample as comparison of rice genotypes), ripening stage on 94 DAT, and maturing stage on 124 DAT. Soil adhering to the stems of the rice plant was then carefully removed by hand using tap water to eliminate algal activity (Watanabe et al., 1978). Furthermore, two cut stems from each plant were used for measuring the ARA. The other two stems were flash-frozen in liquid N and stored at −80°C until freeze-dried. After freeze-drying, the samples were ground with an automatic pulverizer (Automill TK-AM5; Tokken Inc., Chiba, Japan) to determinate NSC and DNA extraction.

### Shading Experiment

The shading experiment was conducted in 2018 at a greenhouse in the Nagoya University. Nipponbare and 2 mutant lines of Nipponbare (*agpl1* and *lse1*) were grown in 12 pots for each genotype. Twelve seedlings were then transplanted to form a circle in a 15,000 a^−1^ Wagner's pot filled up with 3 kg soil from the Togo field. The contents of C and N of the soil were 0.43 and 0.05%, respectively. The fertilizers were then applied at the rates of 0.3 g P_2_O_5_, 0.3 g K_2_O, and 0.5 g N (LP40: LPS100: LP140 = 1:1:1). Subsequently, the pots were submerged at a 3 cm depth with well water throughout the growth periods. Until sampling, newly emerged tillers from the main stem were cut with scissors from the stem base, and only the main stem from each plant was retained.

Shading treatment started at 56 DAT (0 days after shading, DAS) on August 7, 2018 at the booting stage. A mesh cloth was then used to surround the 4 pots of each genotype in the shading treatment. Next, an average cumulative photosynthetically active radiation (PAR) of 23.2 mol m^−2^ day^−1^ was used for the control, whereas a PAR of 0.77 mol m^−2^ day^−1^ was used for shading treatment, respectively. In the control, four pots of each genotype were sampled at 0 and 7 DAS, respectively. However, in the shading treatment, four pots of each genotype were sampled at 6 DAS. Only *agpl1* began heading during 0–7 DAS.

From each pot, four stems were collected, and the basal part was cut into 10 cm lengths. Two of the cut stems were then used for measuring ARA, whereas the other two were flash-frozen in liquid N and stored at −80°C until freeze-dried. Freeze-dried stems subsequently intended for determining NSC were ground with an automatic pulverizer.

### Nitrogenase Activity Estimated by ARA

Two stems from one plant were enclosed in a glass test tube (160 ml) sealed with a silicone lid. The 10% (v/v) of the gas phase in the test tube was then replaced with acetylene, after which the plants were incubated for 24 h in the dark at 25°C. The ethylene concentration was determined using gas chromatographs: GC-2014 (SHIMADZU co., Kyoto, Japan) equipped with a Porapak N 50–80 mesh and a flame ionization detector (FID) connected to Chromatopac Integrator C-R8A (Shimadzu Corporation, Kyoto, Japan) in 2017, and GC-4000 (GL Sciences Inc., Tokyo, Japan) equipped with a Porapak N 80–100 mesh and an FID connected to a Chromato Logger (LC Science Corporation., Nara, Japan) in 2018. The plant sample after ARA measurement was then dried at 80°C for 48 h, and the dry weight was measured. The values of ARA were calculated on the basis of the dry weight measured.

At least one glass test tube without acetylene replacement was prepared for each sampling, and the formation of endogenous ethylene was measured. Since the amount of endogenous ethylene was so small and that it was difficult to detect, ethylene generated as a plant hormone was ignored in this study.

### Determination of NSC

The method described by Sugiura et al. (2015) was followed with slight modifications to determine the NSC in the stems. Briefly, soluble sugars were extracted with 80% ethanol at a temperature of 78.5°C, concentrated centrifugally for 2–4 h, and dissolved in distilled water containing 99% of chloroform. The solution was then incubated in an invertase solution (095-02112; FUJIFILM Wako Pure Chemical Co., Osaka, Japan). Additionally, precipitation-containing starch was heated to 98°C for 1 h and treated with amyloglucosidase (A9228-1G; Sigma-Aldrich, St. Louis, MO, USA) in a 50 mM Na-acetate buffer for 1 h. Next, the glucose concentration and glucose equivalents of sucrose and starch were quantified using the Glucose CII test kit (FUJIFILM Wako Pure Chemical Co., Osaka, Japan), after which the concentration of glucose, sucrose, and starch was calculated on the basis of their dry weight. Total sugar was also calculated by summing glucose and sucrose.

### Determination of N and Carbon Contents

Freeze-dried samples of stems sampled during field experiments were weighed into a tin capsule. Then, the total carbon (C) and N contents were determined using the Vario EL III elementary analyzer (Elementar Co., Hanau, Germany).

### DNA Extraction

The cetyl trimethylammonium bromide (CTAB) protocol was used to extract the total DNA following the method by Doyle and Doyle (1990). Nipponbare, *agpl1*, Leafstar, and CG14, which showed different ARA and NSC concentrations in 2018, were selected for amplicon analysis. Of the four replicates, three that were close to the average of ARA were used. Subsequently, freeze-dried samples were ground and incubated in a 2× CTAB buffer [2% CTAB; 1.4 M NaCl; 20 mM ethylenediamine tetraacetic acid (EDTA); 100 mM Tris HCl pH 8.0] at 65°C for 30 min. Treatment with one volume of chloroform, precipitation with 2-propanol, washing with ethanol (70%), and dissolution in Tris-EDTA (TE) buffer (10 mM Tris, 1 mM EDTA, pH 7.6) was then used to extract the DNA.

### Amplicon Sequencing of the 16S rRNA and *nifH* Genes

The first PCR of the V3–V4 region of bacterial 16S rRNA was conducted using the following blocking primers; the forward primer: 1st-341f_MIX (5′-ACACTCTTTCCCTACACGACGCTCTTCCGATCTNNNNNCCTACGGGNGGCWGCAG-3′) and the reverse primer: 1st-805r_MIX (5′-GTGACTGGAGTTCAGACGTGTGCTCTTCCGATCTNNNNNGACTACHVGGGTATCTAATCC-3′). The first PCR reaction of the *nifH* gene was then conducted using the PolF/PolR primers (Poly et al., 2001); forward primer (5′-TGC GAY CCS AAR GCB GAC TC-3′) and the reverse primer (5′-ATS GCC ATC ATY TCR CCG GA-3′). The PolF/PolR primer set was widely applied previously in N-fixing microbial flora analysis of rice paddy soil (Liao et al., 2018; Ma et al., 2019) and rice endophyte (Banik et al., 2016). The expected amplicon fragment size with PolF/PolR was 362 bp covering positions 115–476 in the *Azotobactervinelandii* reference sequence of *nifH* (M20568.1) (Gaby and Buckley, 2012). Similarly, the first PCR mixture of the 16S rRNA gene and *nifH* gene consisted of 1.0–μl 10 × reaction buffer, 0.8 μl of 2.5 mM deoxyribonucleotide triphosphates (dNTPs), 0.5 μl of 10 μM primers (each), 1.5 μl of 25 μM anti-plastid peptide nucleic acid (PNA), 1.5 μl of 25 μM anti-mitochondrial PNA, 2.0 μl template DNA (0.5 ng μl^−1^), 0.08 μl Ex Taq enzyme (5 U μl^−1^) (Takara Bio Inc., Shiga, Japan), and 2.1 μl milli-Q water. The thermal conditions for the first PCR for both primers were as follows: 95°C for 45 s; 30 cycles of (95°C for 15 s, 78°C for 10 s, 55°C for 30 s, and 72°C for 30 s); and 72°C for 5 min.

Amplicon sequencing of the 16S rRNA and *nifH* genes was then performed by Bioengineering Lab. Co. (Kanagawa, Japan). Here, amplicons were purified using AMPure XP magnetic beads (Beckman Coulter, CA, USA). Subsequently, PCR was conducted again following the instructions of the manufacturer. Sequencing was also conducted on an Illumina MiSeq system (Illumina Inc., San Diego, CA, USA). The MiSeq was run using the 2 × 300 cycle configuration; however, one of the CG14 replicates in the 16S rRNA gene sequence had no amplification, thereby resulting in two replicates. Raw data were defined as raw tags after removing the barcode and PCR primer sequences using the fastq_barcode_splitter from the Fastx Toolkit software (http://hannonlab.cshl.edu/fastx_toolkit/). Next, raw tags were filtered into high-quality (values >20) clean tags using the sickle tools (https://github.com/ucdavis-bioinformatics/sickle).

Pair-end sequences with a length of more than 150 bp for the sequences of the 16S rRNA gene and 40 bp for those of the *nifH* gene, respectively, were selected for the analyses. The reads were then merged using the pair-end merge script FLASH software (http://ccb.jhu.edu/software/FLASH/). Chimera checks were also conducted using USEARCH (Edgar, 2010) and the Greengenes 16S rRNA databases (DeSantis et al., 2006), then clustered into operational taxonomic units (OTUs) with a ≥ 97% nucleotide sequence identity in QIIME (http://qiime.org/tutorials/chimera_checking.html). The sequences of the *nifH* gene were also clustered into OTUs having a ≥97% nucleotide sequence identity using USEARCH as described by Nishihara et al. (2018).

Singleton sequences were removed from the obtained OTUs. For 16S rRNA amplicon data, sequences classified as archaea or chloroplasts were also removed. Then for the *nifH* gene amplicon data, sequences were translated into amino acid sequences, and sequences without Cys 97 and Cys 132 amino acids (protein numbering for NifH protein in *A. vinelandii*; and 4Fe-4S iron-sulfur cluster ligating cysteines) (Howard et al., 1989) were removed.

To assess the similarity among microbial community profiles of each rice genotype, the bacterial community structure analysis was conducted with a non-metric multidimensional scaling (NMDS) using the Bray–Curtis similarity algorithm. Using the R package vegan 2.5–6 (Oksanen et al., 2019) as described by Sasaki et al. (2013), NMDS was calculated from the weighted UniFrac base matrix. As the resulting plots were located close together within each rice genotype, the relative abundance of each OTU of both 16S rRNA and NifH was used as the mean of genotypes in subsequent analyses. Shannon Index (log base 2) and Simpson's evenness were also calculated using the R package vegan.

Further, OTUs having a relative abundance of at least 1% in any rice genotypes were selected for analyses with the 16S rRNA and NifH sequence data, respectively. Phylogenetic analysis for the 16S rRNA gene was also conducted using the SILVA database (SILVA SSU Ref NR_99 database) and the backbone tree (tree_SSURefNR99_1200slv_128). Furthermore, phylogenetic analysis for the NifH was conducted with close relatives as identified by a BLASTp (for NifH) analysis. Sequences were also described as N-fixing bacteria in the nifH_database_2012 (Gaby and Buckley, 2014) using the ARB program (Ludwig et al., 2004). For constructing phylogenetic trees, 32 and 39 obtained OTUs were used to map the 16S rRNA and NifH sequences, respectively. The alignment of all 16S rRNA genes and NifH sequences was done using CLUSTAL W program at the European Bio Informatics server (European Bioinformatics Institute, Cambridge, UK), with the default settings implemented in MEGAX (Kumar et al., 2018). Subsequently, phylogenetic trees were constructed using the maximum-likelihood method by adopting the Tamura–Nei model (Tamura and Nei, 1993) for 16S rRNA sequences, whereas the maximum-likelihood method was executed following the Le_Gascuel_2008 model (Le and Gascuel, 2008) for NifH sequences. The robustness of the tree topologies was tested with 1,000 bootstrap replicates. Here, 1,593 positions (for 16S rRNA genes), and 303 positions (for NifH) were used in the final dataset. Reference type strains are indicated with superscript “†.” As an outgroup, the archaea *Methanosarcinabarkeri* DSM 800^†^ (NR 025303.1) in the tree of the 16S rRNA sequences, and *Roseiflexuscastenholzii* DSM 13941^†^ (GCA 000017805.1), which belongs to the NifH Cluster IV in the tree of NifH sequences, was used. Branches of the phylogenetic tree that consisted of the same class or NifH clusters were grouped in the class or in NifH clusters following the definition of Zehr et al. (2003). Simultaneously, BLASTn (for 16S rRNA sequences) or BLASTp (for NifH sequences) analysis was used to identify close relatives of representative OTUs with ≥1%. To verify the taxonomic affiliation of NifH-OTUs, amino acid sequences were estimated using the BLASTp searches against the non-redundant protein sequence (nr) database and nifH_database_2012 (Gaby and Buckley, 2014). If the closest sequence identity was <90%, it was assigned as an uncultured bacterium.

### Nucleotide Sequence Accession Numbers

Sequence information has been deposited in DDBJ/EMBL/GenBank under the BioProject accession number; PRJDB11557 (PSUB014768), and BioSample accession numbers SAMD00298569–SAMD00298571 (SSUB017990) for Nipponbare, SAMD00298572–SAMD00298574 (SSUB017991) for *agpl1*, SAMD00298575–SAMD00298577 (SSUB017992) for Leafstar, and SAMD00298578–SAMD00298580 (SSUB017993) for CG14. The raw sequences have also been deposited in the DDBJ Sequence Read Archive database under the accession numbers DRA011891 (Nipponbare), DRA011892 (*agpl1*), DRA011893 (Leafstar), and DRA011894 (CG14) for the 16S rRNA gene sequences, and DRA011895 (Nipponbare), DRA011896 (*agpl1*), DRA011897 (Leafstar), and DRA011898 (CG14) for the *nifH* gene sequences. Sequences used to construct the phylogenetic trees have been deposited in DDBJ/EMBL/GenBank with accession numbers from LC628750–LC628781 for 16S rRNA and LC628782–LC628820 for NifH.

### Statistical Analyses

The Tukey–Kramer HSD was used to examine the genotypic and seasonal differences. Spearman's correlation test was used to evaluate the relationship between ARA and NSC concentrations and independent ARA and N concentration. Significant shading treatment effects were explored with Dunnett's test, using 0 DAS as the control. Significant differences were determined at *P* < 0.05. All statistical analyses were conducted using JMP Pro v.15.1.0 (SAS Institute Inc., Cary, NC, USA).

## Results

### ARA, NSC, and N Concentrations in Field-Grown Rice in 2017 and 2018

**Figure 1:**
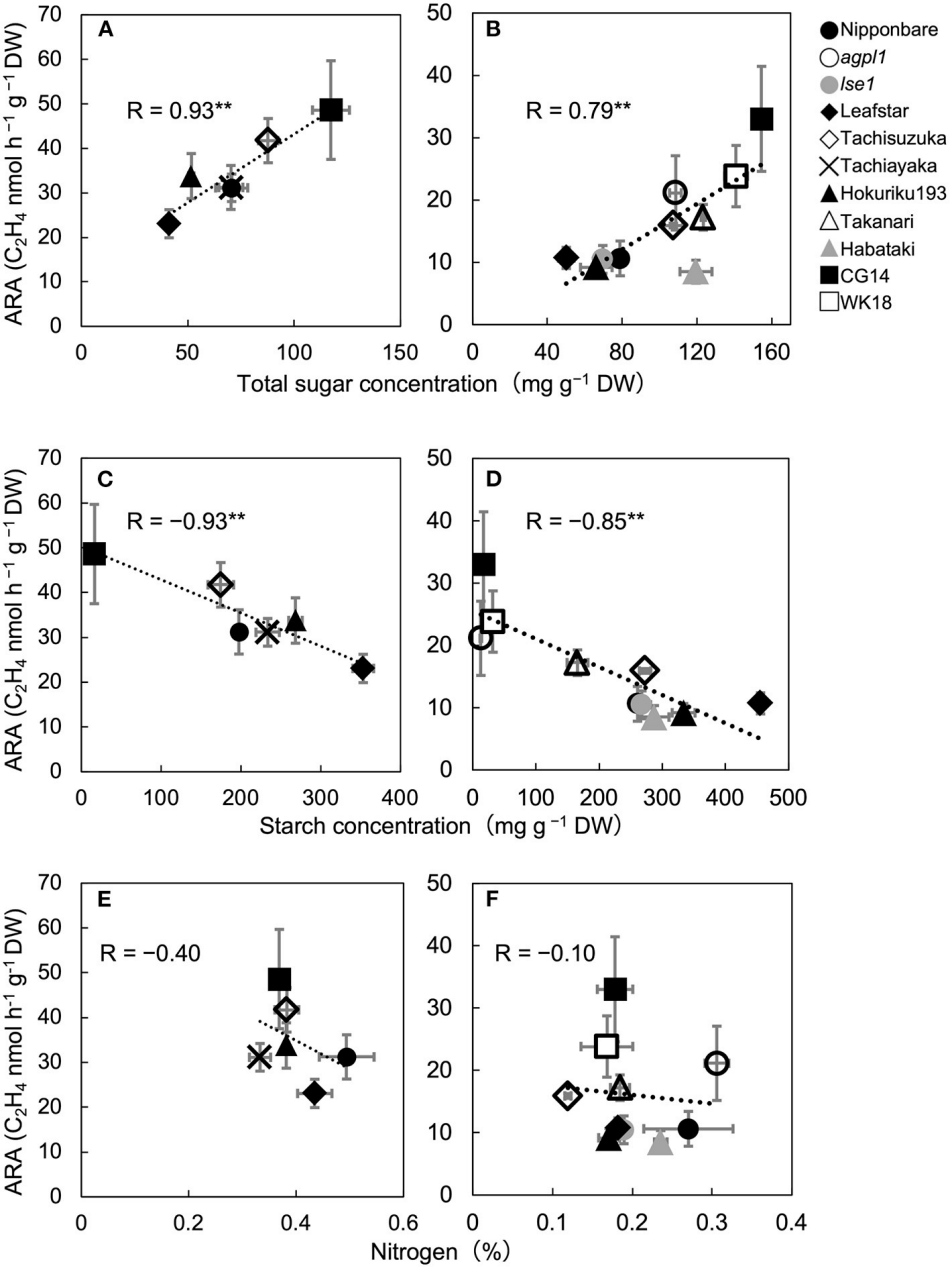
Relationship between acetylene reduction activity (ARA) and total sugar (sucrose and glucose) concentration of stem in six rice varieties in 2017 **(A)** and 10 rice genotypes in 2018 **(B)**, correlation between ARA and starch concentration in 2017 **(C)** and 2018 **(D)**, and correlation between ARA and nitrogen concentration in 2017 **(E)** and 2018 **(F)**, at heading stage in the field experiment. The data for non-structural carbohydrates (glucose, sucrose, and starch) were used from Table 2. Data are means ± SE (*n* = 3 in 2017 and *n* = 4 in 2018). **indicates significance at *P* = 0.01.

The acetylene reduction activity varied widely among varieties and was the highest in CG14 in both 2017 and 2018 (Figure 1). The NSC concentrations in the field experiments are indicated in Table 2. For sugars, sucrose showed a higher concentration than glucose in all rice genotypes for both years. The sucrose concentration was the highest in cv. CG14 and lowest in cv. Leafstar for both years, respectively. Similarly, the glucose concentration was the highest in CG14 in 2017 and cv. WK18 in 2018. Starch concentration was also the highest in Leafstar for both years and the lowest in CG14 in 2017, and rice genotypes, *agpl1*, CG14, and WK18 in 2018.

**Table 2:** Non-structural carbohydrate (NSC) concentrations and nitrogen (N) concentration of stem of various rice genotypes at heading stage in 2017 and 2018.

	**2017**			**2018**		
**Rice genotypes**	**NSC concentration (mg g** ^****−1****^ **DW)**			**NSC concentration (mg g** ^****−1****^ **DW)**		
	**Glucose**	**Sucrose**	**Starch**	**Glucose**	**Sucrose**	**Starch**
Nipponbare	5.53 ± 0.4b	64.7 ± 6.0bc	197.5 ± 4.4cd	2.65 ± 0.1b	76.3 ± 1.9c	260.8 ± 1.9c
*agpl1*	-	-	-	4.56 ± 0.2b	103.9 ± 3.2bc	13.0 ± 1.3e
*lse1*	-	-	-	2.64 ± 0.1b	66.9 ± 4.2d	267.6 ± 14.0c
Leafstar	4.85 ± 0.1b	36.4 ± 0.9c	353.3 ± 13a	0.88 ± 0.3b	49.2 ± 3.9d	455.4 ± 3.9a
Tachisuzuka	5.63 ± 0.4b	82.2 ± 4.2ab	233.5 ± 15bc	2.08 ± 1.2b	105.2 ± 4.3bc	272.8 ± 4.3bc
Tachiayaka	7.12 ± 1.3b	63.5 ± 8.3bc	174.8 ± 16d	-	-	-
Hokuriku193	5.61 ± 0.1b	46.0 ± 1.4c	268.4 ± 8.4b	13.09 ± 9.9ab	53.0 ± 14.5d	333.7 ± 14.5b
Takanari	-	-	-	5.18 ± 0.6b	118.1 ± 1.7ab	165.8 ± 1.7d
Habataki	-	-	-	4.18 ± 0.5b	115.2 ± 8.4ab	287.3 ± 8.4bc
CG14	14.73 ± 1.9a	102.6 ± 9.7a	16.2 ± 4.5e	13.80 ± 2.7ab	140.7 ± 7.0a	17.3 ± 7.0e
WK18	-	-	-	23.57 ± 2.0a	117.3 ± 7.8ab	32.2 ± 7.8e

*Data are means ± SE (n = 3 in 2017; n = 4 in 2018). Significant differences were determined at P <0.05 using Tukey–Kramer honestly significant difference (HSD)*.

The mean ARA and total sugar concentration of each rice genotype were positively correlated during both years (Figures 1A,B). The correlation coefficient between ARA and sucrose concentration and that between ARA and glucose concentration were R = 0.74 (*P* = 0.090) and 0.86 (*P* = 0.029) in 2017 and 0.74 (*P* = 0.013) and 0.56 (*P* = 0.093) in 2018. The ARA and starch concentration were also negatively correlated in both the years (Figures 1C,D). Comparisons among cv. Nipponbare and its two mutants showed that *agpl1* was the highest in ARA association with the highest sugar and lowest starch concentrations (Figures 1B,D).

Also, N concentration varied from 0.37 to 0.49% and 0.12 to 0.31% in 2017 and 2018, respectively. No significant correlation was found between ARA and N concentration among rice genotypes in both the years (Figures 1E,F).

### Changes in ARA, NSC, and N Concentrations in Nipponbare at Different Growth Stages in 2018

**Figure 2:**
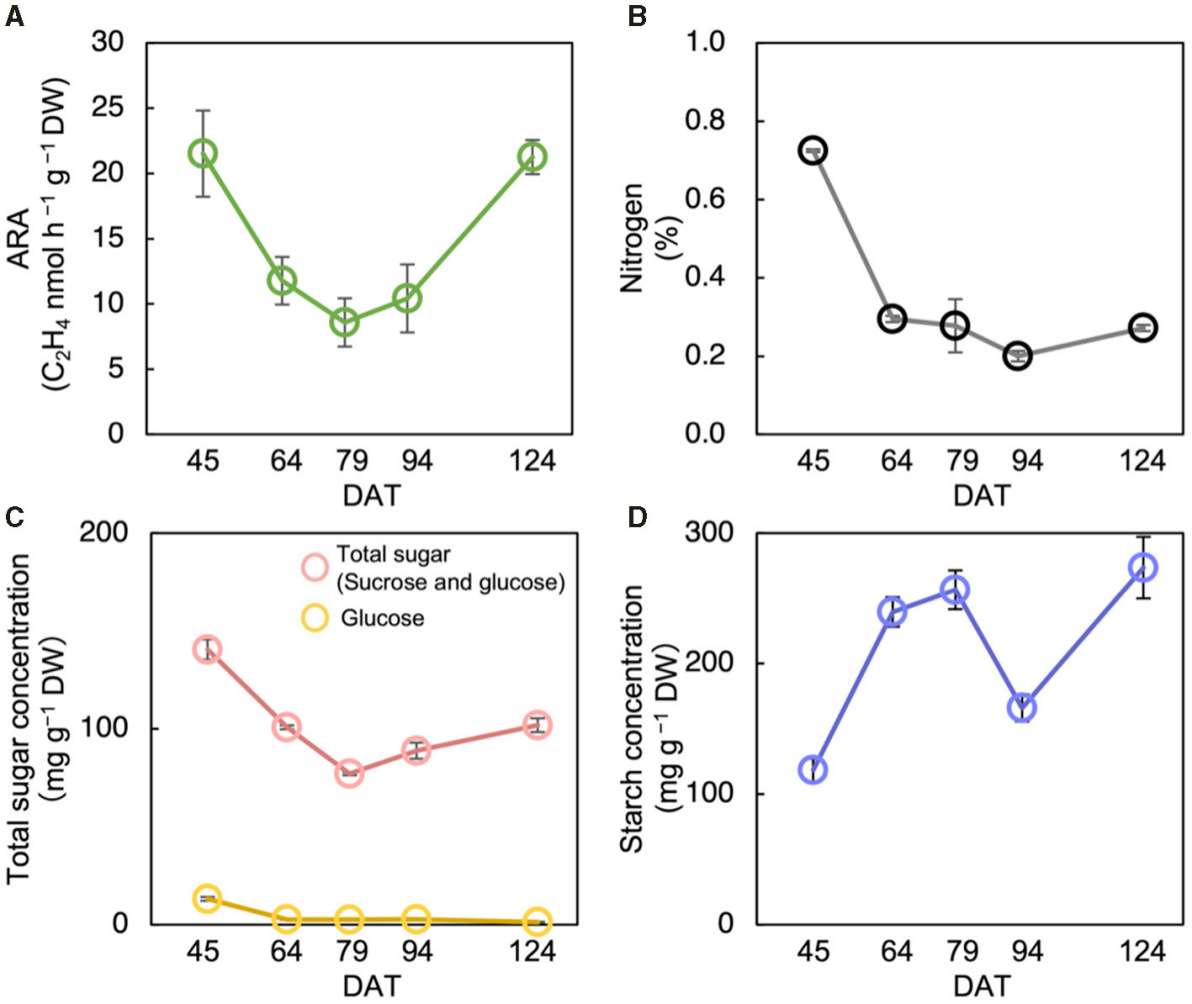
Changes in acetylene reduction activity (ARA) **(A)**, nitrogen concentration **(B)**, total sugar (sucrose and glucose) concentration **(C)**, and starch concentration **(D)** of stem at different growth stages in Nipponbare at field experiment in 2018. Data for Nipponbare in Table 2 was used for the data at 79 day after transplanting (DAT). The growth stages were the tillering stage on 45 DAT, panicle initiation stage on 64 DAT, heading stage on 79 DAT, ripening stage on 94 DAT, and the maturing stage on 124 DAT. Data are means ± SE (*n* = 4 for 79 DAS, *n* = 3 for 45, 64, 94, and 124).

The acetylene reduction activity in Nipponbare decreased until the heading stage and increased again at ripening (Figure 2A). The N concentration decreased significantly from the tillering stage to the heading stage, and then did not change significantly (Figure 2B). Total sugar concentration decreased until the heading stage and increased until the maturing stage (Figure 2C). Similarly, starch concentration increased until the heading stage, decreased during the ripening stage, after which it increased until the maturing stage (Figure 2D). The ARA and total sugar concentration were changed almost synchronously, notably from the tillering stage to the ripening stage (Figures 2A,C).

### Shading Experiment

**Figure 3:**
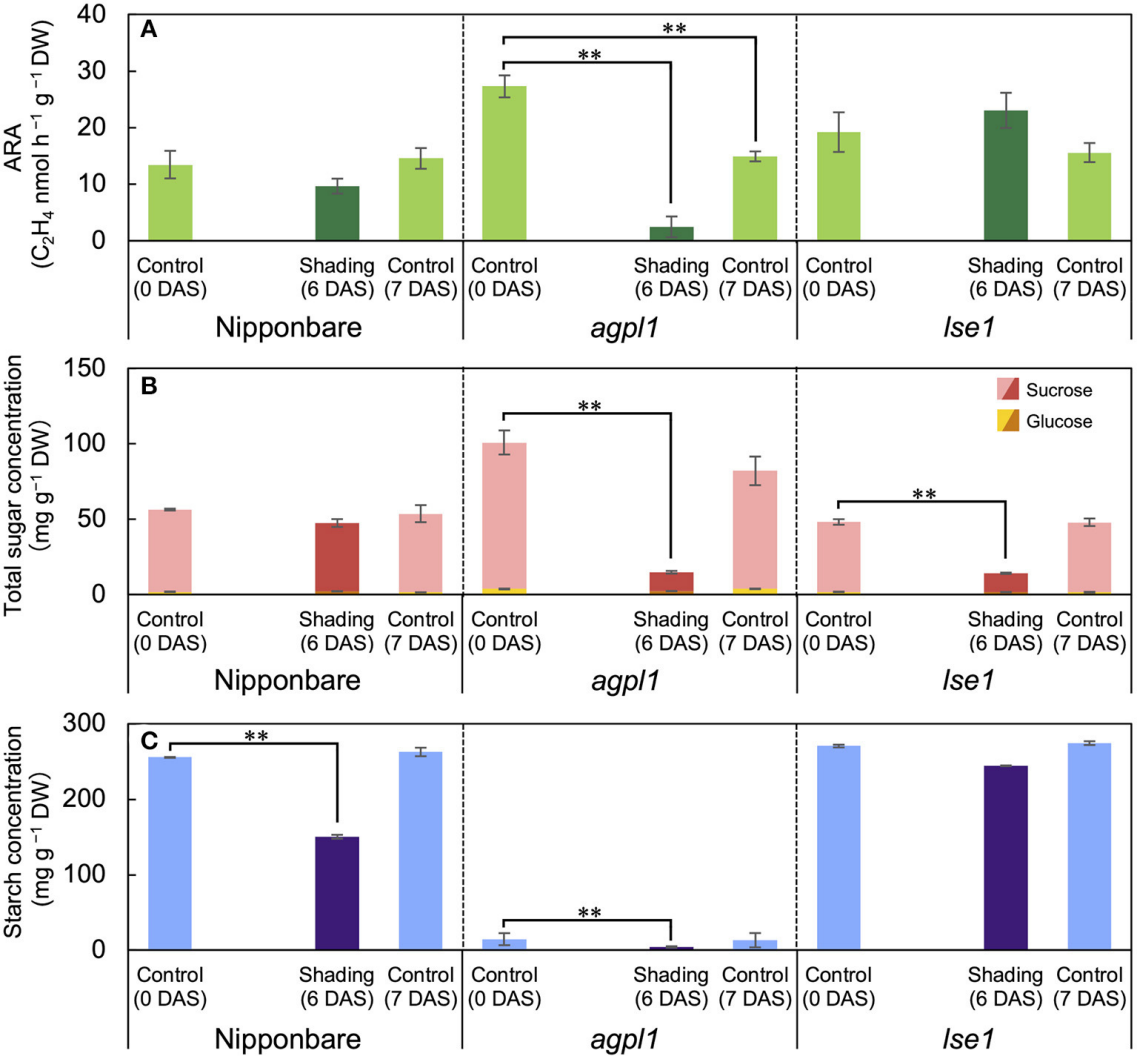
The acetylene reduction activity (ARA) **(A)**, total sugar (sucrose and glucose) concentration **(B)**, and starch concentration **(C)** in stem of rice genotypes at shading experiment. DAS indicates days after shading. Data are means ± SE (*n* = 4). **indicate significance at *P* = 0.01 level assessed by Dunnett's test. In the Dunnett's test, values of 0 DAS served as control. Sugars were tested at the total sugar concentration of sucrose and glucose.

The acetylene reduction activity and total sugar concentration of *agpl1* was about two times as large as that of Nipponbare in the control before the shading treatment (Figures 3A,B). However, the starch concentration of *agpl1* was more than ten times lower than that of Nipponbare and rice genotype, *lse1* (Figure 3C).

The shading treatment reduced the total NSC (glucose, sucrose, and starch) concentration of Nipponbare, *agpl1*, and *lse1* by 114 mg g^−1^ DW (37%), 96 mg g^−1^ DW (83%), and 60 mg g^−1^ DW (19%), respectively, than those of the control at 0 DAS. The shading treatment significantly decreased the starch concentration in Nipponbare compared to the control at 0 DAS, and the ARA and total sugar concentration also decreased, although not significantly (Figures 3A,B). The ARA of *agpl1* was greatly reduced by 91% with shading treatment accompanied by the decrease in total sugar and starch concentrations, and the no shading at 7 DAS also showed a decrease in ARA (Figure 3). In *lse1*, the total sugar concentration was reduced by shading without a significant reduction in ARA (Figures 3A,B). The starch concentration of *lse1* was also comparable to that of Nipponbare in the control before shading (0 DAS), but unlike Nipponbare, it did not decrease significantly during the shading treatment (Figure 3C).

### Bacterial Community Structure and Composition in 16S rRNA Gene

**Figure 4:**
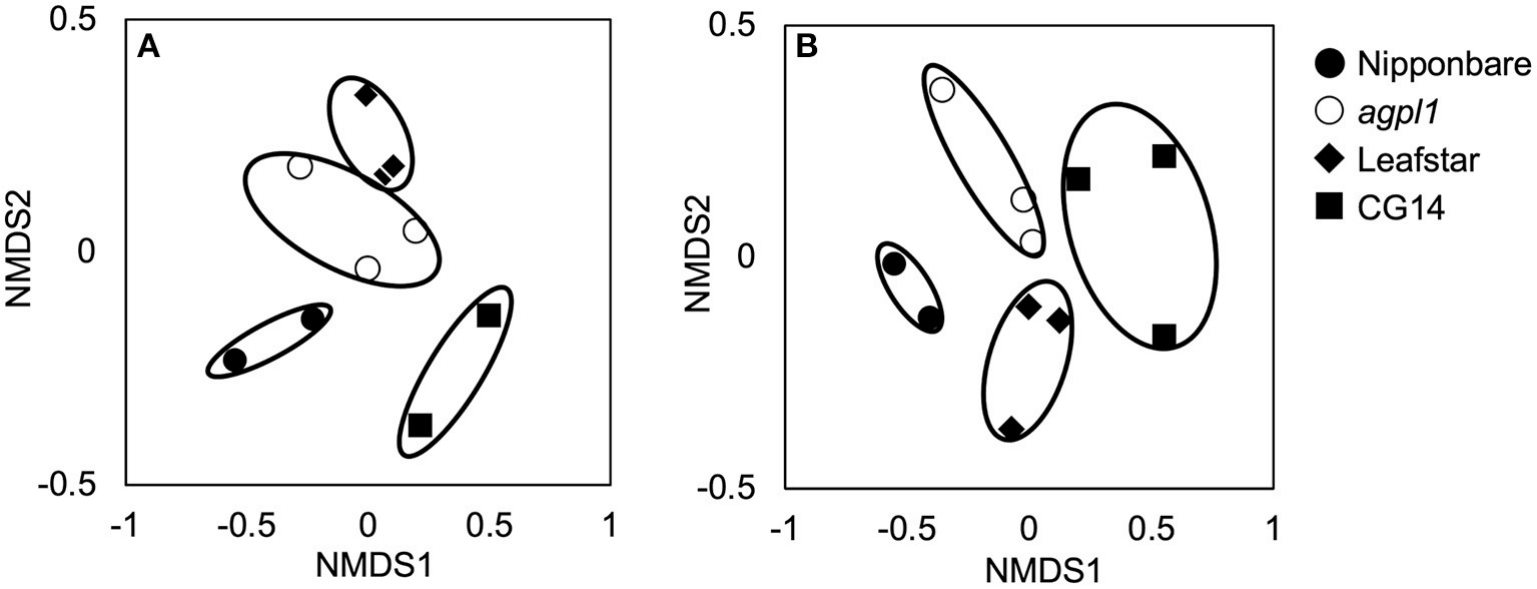
Non-metric multidimensional scaling (NMDS) plots of 16S rRNA libraries **(A)** and NifH libraries **(B)** of bacterial communities in stem of four rice genotypes at the heading stage, comparing community dissimilarities (based on Bray–Curtis distances) in the 2018 field experiment among each replicate (*n* = 2 or 3). Replication of each genotype (*n* = 2 or 3) are indicated for each symbols.

Totally 3,583–20,126 reads and 611–1,757 OTUs were obtained from the 10 samples using the amplification of the 16S rRNA genes (*n* = 3 for *agpl1* and Leafstar, *n* = 2 for Nipponbare and CG14) (Supplementary Table 1). Also, since the analysis of 16S rRNA sequences revealed that one of the replicates of Nipponbare was infected with a plant pathogen, *Pantoeaananatis*, the replicate and the corresponding *nifH* replicates of Nipponbare were removed from the analysis. The NMDS plot also showed that four rice genotypes were separated from each other based on the 16S-OTU compositions (Figure 4A). The 16S rep1 had less reads than others, but it was included in the subsequent analyses since its NMDS value was close to that of 16S rep2. The Shannon diversity index, however, did not vary much among rice genotypes (Supplementary Table 1).

**Figure 5:**
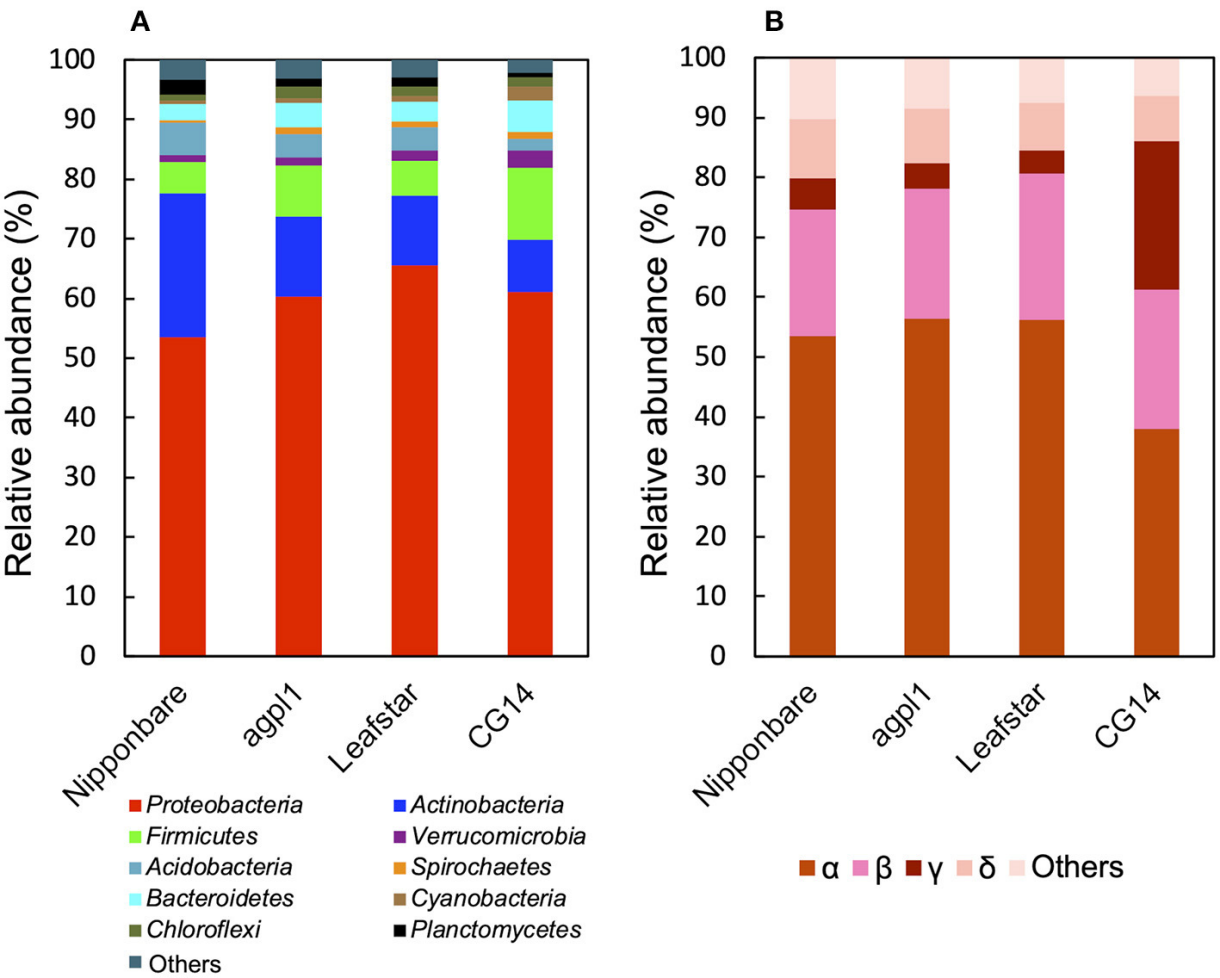
Relative abundance of the phylum **(A)**, subdivision of *Proteobacteria* **(B)** based on 16S rRNA gene sequence in the stems of four rice genotypes at the heading stage in field experiment in 2018. Mean relative abundance of the 16S-OTUs in each rice genotype was used for calculations (*n* = 2 for Nipponbare and CG14; *n* = 3 for *agpl1* and Leafstar). The letters α, β, γ, and δ indicate alpha, beta-, gamma-, and delta-proteobacteria, respectively.

Based on the 16S rRNA sequence analysis, the relative abundance of sequences in each sample is classified at the phylum level (Figure 5A). Members of the phylum *Proteobacteria* were the most dominant in all rice genotypes (53.4–65.5% of the total reads) (Figure 5A). Furthermore, the classification by class levels revealed that *Alphaproteobacteria* was the most prevalent in the phylum, *Proteobacteria* for all rice genotypes (37.9–56.5% of the total reads classified as *Proteobacteria*) (Figure 5B). Also, the three rice genotypes other than CG14 had the highest abundance of *Betaproteobacteria* followed by *Alphaproteobacteria*, whereas CG14 had the second-highest amount of *Gammaproteobacteria* with an abundance ratio of 24.9% during the total reads of the phylum classified as *Proteobacteria*. This result was much higher than the other three rice genotypes showing 2.7–3.1% (Figure 5B).

Other phyla detected in the communities by the 16S rRNA gene sequence analysis include *Acidobacteria, Actinobacteria, Bacteroidetes, Chloroflexi, Cyanobacteria, Firmicutes, Planctomycetes, Spirochaetes*, and *Verrucomicrobia* (Figure 5A). The abundance of the phylum, *Actinobacteria* was the second-highest in all rice genotypes except for CG14 in which the phylum, *Firmicutes* was the second-highest (Figure 5A).

**Figure 6:**
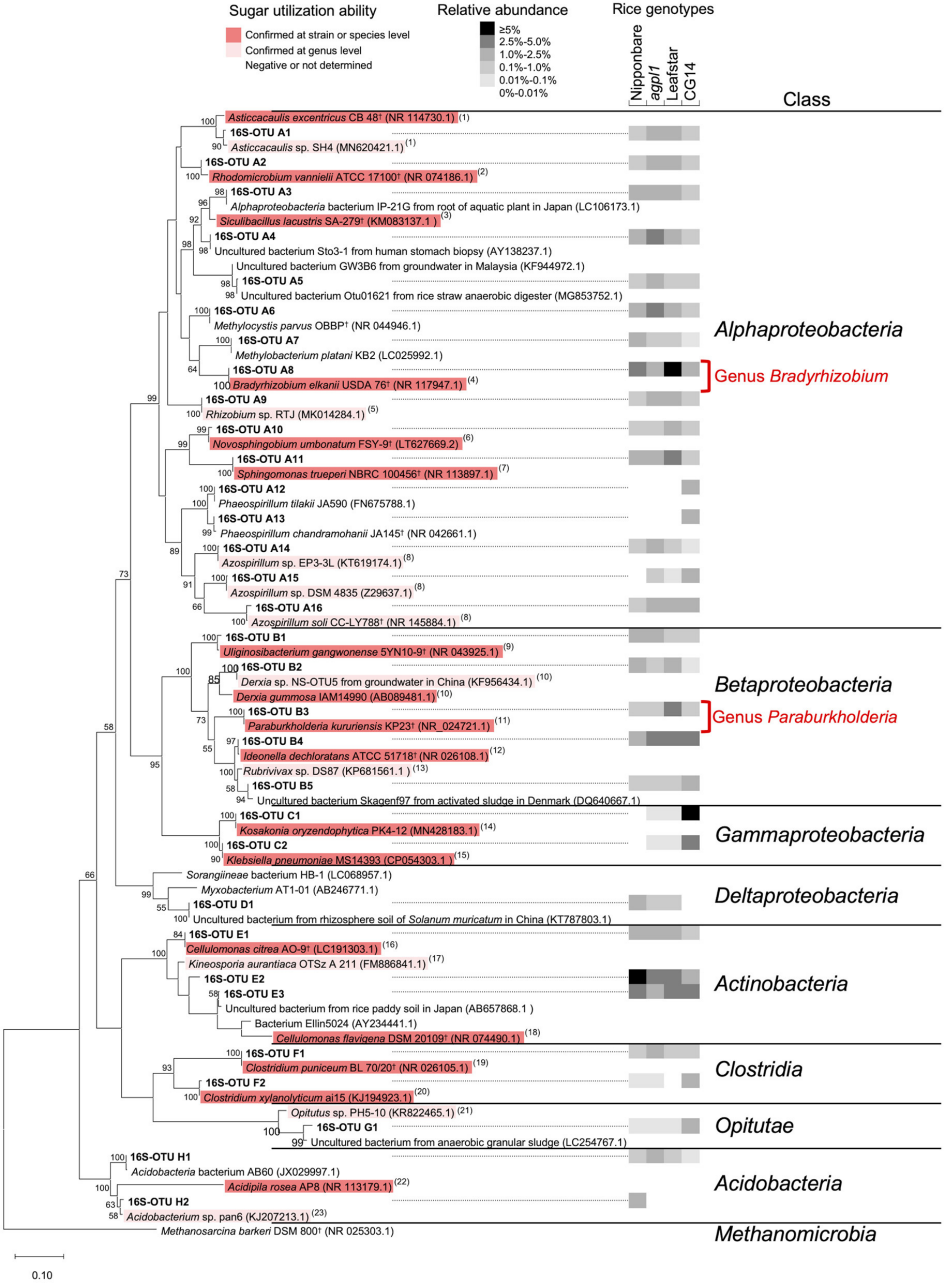
Maximum-likelihood phylogenetic tree of 16S rRNA sequences obtained from the stems of four rice genotypes and amplified by V3–V4 region of 16S rRNA primer set. Operational taxonomic units (OTUs) with a mean relative abundance of ≥1% in at least one rice genotype were used. Bootstrap values of ≥50% are indicated at the respective nodes. Reference type strains are indicated with superscript “†.” The backgrounds of the reference strains are colored according to the sugar (sucrose and/or glucose) utilization capacity of those bacteria based on the data from (1) Poindexter (2015); (2) Hamzah et al. (2006); (3) Felföldi et al. (2019); (4) Sajnaga and Jach (2020); (5) Kuykendall et al. (2015); (6) Sheu et al. (2020); (7) Xie and Yokota (2006); (8) Raj et al. (2012); (9) Weon et al. (2008); (10) Kennedy (2015); (11) Anandham et al. (2009); (12), Noar and Buckley (2009); (13) Imhoff (2015a); (14) Hardoim et al. (2013); (15) Grimont and Grimont (2015); (16) Lee et al. (2020); (17) Normand and Benson (2015); (18) Stackebrandt and Kandler (1979); (19) Holt et al. (1988); (20), Rainey et al. (2015); (21) Janssen (2015); (22) Okamura et al. (2011); (23) Thrash and Coates (2015). The black-and-white shading in the columns on the right-hand of the 16S-OTU name indicates the mean relative abundance of the 16S-OTUs in each rice genotype (*n* = 2 for Nipponbare and CG14; *n* = 3 for *agpl1* and Leafstar). The 16S rRNA classification was clustered using known sequences.

To examine the taxonomy of predominating bacteria, a maximum-likelihood phylogenetic tree was constructed with 32 representative 16S-OTUs, presenting ≥1% in relative abundance of at least one sample listed in the Supplementary Table 2 (Figure 6). The dominant OTUs with ≥1% the relative abundance accounted for 35% of total OTUs on average (33.0% in Nipponbare, 35.3% in *agpl1*, 37.8% in Leafstar, and 32.0% in CG14). The 16S-OTUs detected in this analysis were also distributed to eight phylogenetic groups, A–H, by class level (Figure 6). Similarly, 10 16S-OTUs were observed with ≥2.5% relative abundance in at least 1 rice genotype. Closely related species with more than 95% identity by BLASTn were A4 from the *Siculibacilluslacustris* strain SA-279^†^ (KM083137.1), A6 from *Methylocystis parvus* OBBP^†^ (NR_044946.1), A8 from *Bradyrhizobium elkanii* USDA 76^†^ (NR 117947.1), A11 from *Sphingomonastrueperi* NBRC 100456^†^ (NR113897.1), and B3 from the *Paraburkholderia kururiensis* strain KP23^†^ (NR_024721.1). Furthermore, B4 from the *Ideonelladechloratans* strain BK-22 (KU360710.1), 16S-OTU C1 from the *Kosakoniaoryzendophytica* strain PK4-12 (MN428183.1), C2 from the *Klebsiella pneumoniae* strain MS14393 (CP054303.1), E2 from the *Kineosporiaaurantiaca* strain OTSz_A_211 (FM886841.1), and E2 from Bacterium Ellin5024 (AY234441.1) were also among the closely related species with more than 95% identity by BLASTn (Figure 6, Supplementary Table 2).

### Bacterial Community Structure and Composition in *nifH* Gene

Amplification was used to obtain 28,394–56,643 reads and 192–516 OTUs from 11 samples by the amplification of the *nifH* gene (*n* = 3 for *agpl1*, Leafstar, and CG14, *n* = 2 for Nipponbare) (Supplementary Table 1). The NMDS plots for the NifH as well as the 16S rRNA were located close together within the same rice genotypes based on the OTU composition (Figure 4B); however, the Shannon diversity index did not vary much among the rice genotypes (Supplementary Table 1).

**Figure 7:**
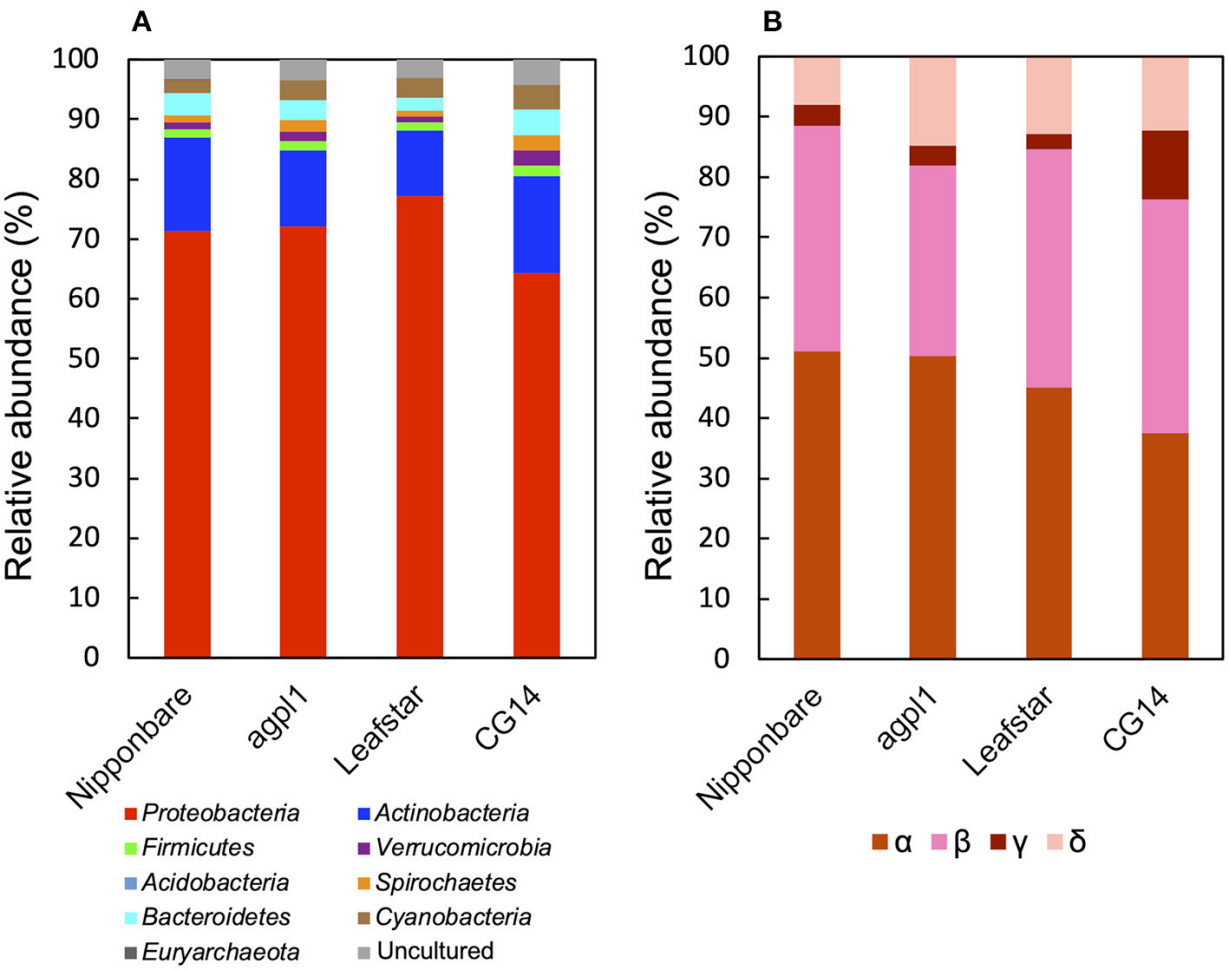
Relative abundance of the phylum **(A)**, subdivision of *Proteobacteria* **(B)** based on NifH sequences obtained from the stems of four rice genotypes at the heading stage during the field experiment in 2018. Mean relative abundance of the NifH-OTUs in each rice genotype were used for calculations (*n* = 2 for Nipponbare; *n* = 3 for *agpl1*, Leafstar, and CG14). The letters α, β, γ, and δ indicate alpha-, beta-, gamma-, and delta-proteobacteria, respectively. Varying taxonomic affiliation of NifH-OTUs, amino acid sequences were BLASTp searches against the non-redundant protein sequence (nr) database for ≥0.5% relative abundance OTUs and nifH_database_2012 (Gaby and Buckley, 2014) for <0.5% relative abundance OTUs. If the closest sequence identity was <90%, it was assigned as an uncultured bacterium.

Furthermore, based on the NifH sequence analysis, *Proteobacteria* was the most dominant bacterial phylum (64.2–77.3% of the total reads) (Figure 7A), which was similar to that of the 16S rRNA.

In phylum *Proteobacteria*, members of the class *Alphaproteobacteria* were the most abundant, followed by the class *Betaproteobacteria* in Nipponbare, *agpl1*, and Leafstar (Figure 7B). Alternatively, in CG14, members of the class *Alphaproteobacteria* and *Betaproteobacteria* had roughly in equal abundance (Figure 7B). Similarly, the class *Deltaproteobacteria* was the third most abundant following the class *Alphaproteobacteria* and *Betaproteobacteria* in all genotypes (Figure 7B). Also, the class *Gammaproteobacteria* was more abundant in CG14 (11.3% of the total reads classified as *Proteobacteria*) than in other rice genotypes (2.4–3.5%) (Figure 7B). The abundance of *Actinobacteria* was the second-highest among all rice genotypes (Figure 7A). Other phyla detected in the communities by the NifH sequence analysis were *Acidobacteria, Bacteroidetes, Cyanobacteria, Euryarchaeota, Firmicutes, Spirochaetes*, and *Verrucomicrobia* (Figure 7A).

**Figure 8:**
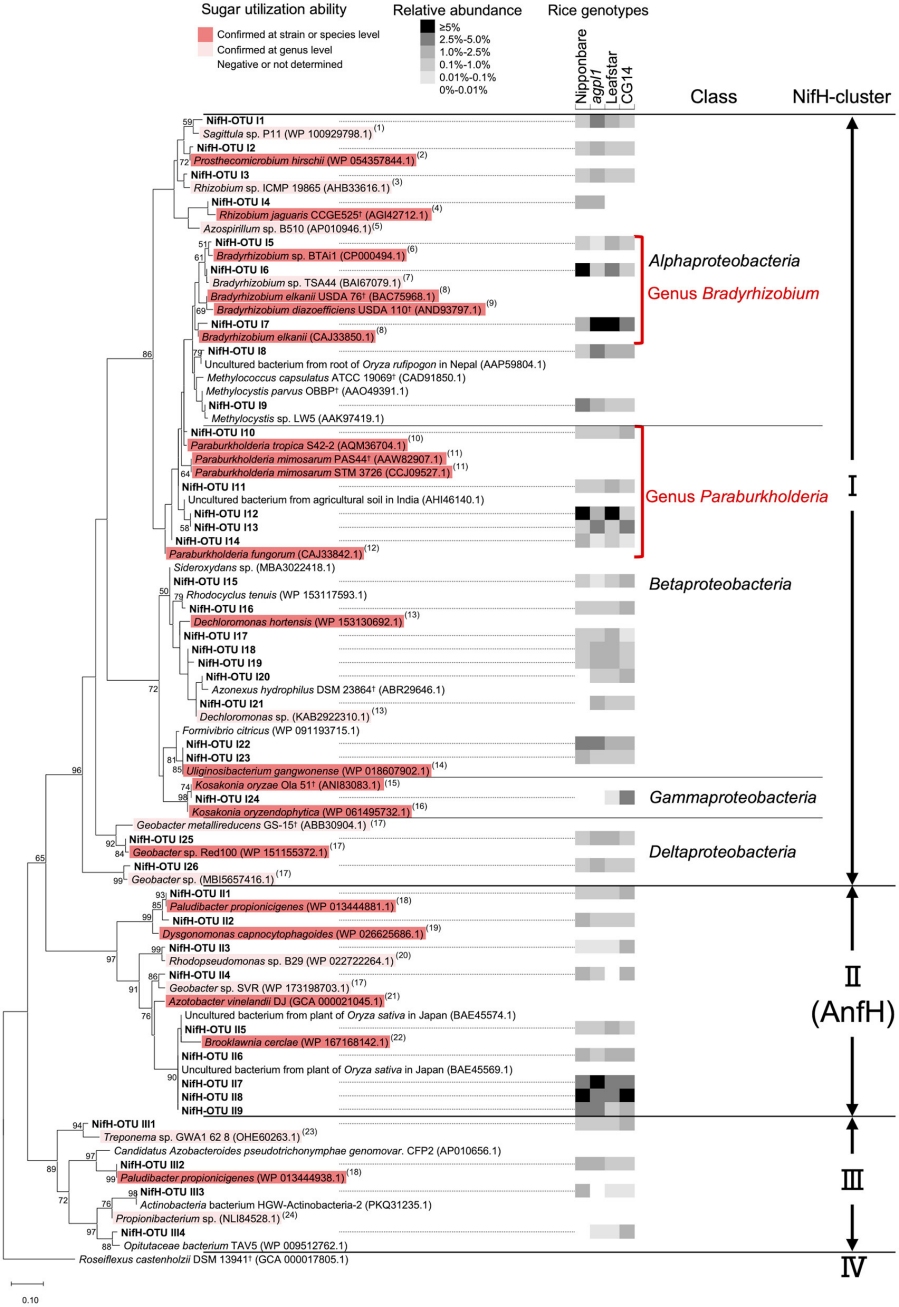
Maximum-likelihood phylogenetic tree of NifH sequences obtained from the stems of four rice genotypes and amplified by the *nifH* primer set. OTUs with a mean relative abundance of ≥1% in at least one rice genotype were used. Bootstrap values of ≥50% are indicated at their respective nodes. Reference type strains are indicated with superscript “†.” The backgrounds of the reference strains are colored according to the sugar (sucrose and/or glucose) utilization capacity of those bacteria based on the data from (1) Lee et al. (2013); (2) Staley (1984); (3) Kuykendall et al. (2015); (4) Dall'Agnol et al. (2013); (5) Baldani et al. (2015); (6) Hungria et al. (1993); (7) Kuykendall (2015); (8) Sajnaga and Jach (2020); (9) Ahnia et al. (2018); (10) Reis et al. (2004); (11) Chen et al. (2006); (12) Coenye et al. (2001); (13) Wolterink et al. (2005); (14) Weon et al. (2008); (15) Qiu et al. (2017); (16) Hardoim et al. (2013); (17) Xu et al. (2020); (18) Ueki et al. (2006); (19) Olsen (2015); (20) Imhoff (2015b); (21) Diaz-Barrera et al. (2016); (22) Bae et al. (2006); (23) Norris et al. (2015); and (24) Patrick and McDowell (2015). The black-and-white shading in the columns on the right-hand of the NifH-OTU name indicates the mean relative abundance of the NifH-OTUs in each rice genotype (*n* = 2 for Nipponbare; *n* = 3 for *agpl1*, Leafstar, and CG14). When the groups were clustered with known sequences from a single class, the class name was added. The NifH cluster classification was defined from I to IV by Zehr et al. (2003) based on the clustering in the phylogenetic tree.

The maximum-likelihood phylogenetic tree constructed with 39 representative NifH-OTUs presented ≥1% relative abundance in at least 1 sample (Figure 8). The dominant NifH-OTUs with ≥1% relative abundance accounted for 45% of the total on an average of the four genotypes (47.0% in Nipponbare, 45.7% in *agpl1*, 44.0% in Leafstar, and 41.0% in CG14). Similarly, NifH clusters were used to distribute the NifH-OTUs detected in this study to three phylogenetic groups (Figure 8). Also, there were 10 NifH-OTUs with ≥2.5% relative abundance in at least 1 rice genotype; NifH-OTU I1 from *Sagittula*sp. P11 (WP_100929798.1), I6 from *Bradyrhizobium* sp. TSA44 (BAI67079.1), I7 from *Bradyrhizobium elkanii* (CAJ33850.1), I8 from *Methylocella tundrae*T4^†^ (CAD91836.1), I9 from *Methylocystis parvus* OBBP^†^ (AAO49391.1), I12 & I13 from *Paraburkholderia mimosarum* STM 3726 (CCJ09527.1), I22 from *Uliginosibacterium gangwonense* (WP_018607902.1), I24 from *Kosakonia oryzae* (ALH07171.1) in Clusters I and II7, and II8 & II9 from *Brooklawnia cerclae* (WP_167168142.1) in Cluster II (Figure 8, Supplementary Table 3).

### Community Analysis of Potential N-Fixing Bacteria

The relative abundance of OTUs from *Bradyrhizobium* sp. (class *Alphaproteobacteria*) and *Paraburkholderia* sp. (class *Betaproteobacteria*) was high in all samples of Nipponbare, *agpl1*, Leafstar, and CG14, and the total relative abundance of NifH representative OTUs (>1% relative abundance in at least one sample) classified as *Bradyrhizobium* sp. (NifH-OTU I5–I7) and *Paraburkholderia* sp. (NifH-OTU I10–I14) were 9.0, 8.0, 12.1, 3.7%, and 9.8, 5.7, 10.7, 6.1%, respectively (Figure 8, Supplementary Table 3). Similarly, in 16S rRNA, the relative abundance classified as *Bradyrhizobium* sp. (16S-OTU A8) and *Paraburkholderia* sp. (16S-OTU A8) were 2.6, 2.1, 5.2, 1.6%, and 0.7, 0.8, 3.7, 0.4% in Nipponbare, *agpl1*, Leafstar, and CG14, respectively (Figure 6, Supplementary Table 2). Five NifH-OTUs closely related to *Brooklawnia* sp. (phylum *Actinobacteria*) also had low amino-acid identity compared to the closest described species (93.4–94.4%). Furthermore, the total relative abundance of the representative sequences (NifH-OTU II5–9) was found to be 13.8, 12.5, 10.3, and 15.4% in Nipponbare, *agpl1*, Leafstar and CG14, respectively (Figure 8, Supplementary Table 3). The sequences matched or closely matched with those of uncultured bacterium (BAE45574.1 or BAE45569.1); however, the corresponding sequences of 16S rRNA were not found in this study.

The NifH-OTU I24 classified as *Kosakonia* sp. (class *Gammaproteobacteria*) was also abundant only in CG14 with a relative abundance of 3.5% (Figure 8, Supplementary Table 3). In 16S rRNA, 16S-OTU C1 classified as *Kosakonia* sp. was <0.06% compared with the relative abundance in Nipponbare, *agpl1*, and Leafstar; however, the relative abundance was 8.74% in CG14 (Figure 6, Supplementary Table 2).

### Sugar Utilization Capacity of Potential N-Fixing Bacteria

For reference strains used in the phylogenetic trees of 16S rRNA and NifH (Figures 6, 8), the sugar (sucrose and glucose) utilization ability was estimated at the strain, species, and at the genus levels with available information. Most of the reference strains selected for the phylogenetic trees of 16S rRNA and NifH were reported to be able to utilize sugars, regardless of the class or NifH- cluster to which they belonged (Figures 6, 8). In *Alphaproteobacteria*, which had high abundance in the class (Figures 5, 7), most bacteria other than methane-oxidizing bacteria were considered to be capable of sugar utilization (Figures 6, 8). The strains of *Bradyrhizobium* sp. (NifH-OTU I5–I7) and *Paraburkholderia* sp. (NifH-OTU I10–I14), which were adjacent to the NifH-OTUs with high relative abundance, were capable to utilize sugars (Figure 8). Strains of *Kosakonia* sp., which had a high abundance in CG14, were also able to utilize sugar (Figures 6, 8).

## Discussion

### Relationship Between N-Fixing Activity and NSC in Rice Stem

Results revealed that N fixation can positively be affected by total sugar concentration rather than by starch or N concentrations. The fact that ARA and total sugar concentration in the stem were positively correlated with rice varieties through the 2 years of experiments in the field supported these results (Figures 1A,B). From the results, *agpl1*, a mutant of Nipponbare that cannot synthesize and store starch in the stem tended to show higher ARA and total sugar concentration than Nipponbare in 2018 (Figure 1B). In Nipponbare, changes in ARA and total sugar concentration also coincided with each other starting from tillering to the ripening stage in 2018 (Figure 2), which also supports the results described above.

The possibility of enhancing endophytic N fixation by the accumulation of sugars in rice stems is consistent with the observations of high contributions of BNF in other crops, such as sugarcane, sweet potato, and pineapple, which have high sugar levels (Lima et al., 1987; Yoneyama et al., 1998; Ando et al., 2000). Carbohydrates produced by photosynthesis are stored in the stem mainly as starch and sucrose in rice (Yoshida, 1972) as shown in this study (Table 2, Figure 2). In the mature stem of sugarcane, about 10–20% of the accumulated sugars were present in the apoplast and contributed to high endophytic BNF (Welbaum and Meinzer, 1990; Dong et al., 1994; Yoneyama et al., 2019). The shading experiment also indicated that changes in sugar are more crucial than starch for the fluctuation of N-fixing activity, since ARA in *agpl1* decreased significantly by shading treatment in association with a large decrease in sugar as compared with Nipponbare (Figure 3).

Considering that most endophytes inhabit the intercellular spaces (Bacon and Hinton, 2006), direct carbon sources for endophytes were from the apoplastic fluid and cell walls. Sugars are mainly stored intracellularly and are present in the apoplast (Scofield et al., 2007), whereas the starch is synthesized and stored in the intracellular chloroplast and amyloplast (Martin and Smith, 1995). Therefore, the close relationship between sugar level and ARA suggested that sugars present in the apoplast are more influential on the N-fixing endophytes than starch. The total sugar concentration in plant tissues may reflect the total sugar concentration in the apoplast, although it is necessary to measure sugar concentrations in the apoplastic fluid separately. In contrast, starch, which accumulates intracellularly unlike sugar, would not have a direct effect on N fixation. Furthermore, the negative correlation between ARA and starch concentration (Figures 1C,D), as well as the opposing relationship between ARA and total sugar concentration (Figures 1A,B), is due to the tradeoff between starch and sugar accumulation. Thus, rice genotypes, such as CG14 that mainly store sugars, are proposed to be advantageous for enhancing N-fixing ability; whereas rice genotypes, such as Leafstar, which have a high capacity to synthesize starch, are proposed to have low N-fixing activity. Leafstar, which accumulated more starch, also showed the same trend as in a previous study (Ookawa et al., 2010). By shading treatment, the reason for less reduction in ARA by the shading of *lse1* was not so clear (Figure 3). The reduction in total NSC (glucose, sucrose, and starch) in *lse1* was about half of that in Nipponbare (Figure 3). Therefore, it can be speculated that *lse1* accumulated much more starch in the leaf than Nipponbare (Hirose et al., 2013), which was degraded and supplied to stems in the form of sugars or organic acids. Even if not directly, starch is the pool that supplies sugar to the endophytes.

The N concentration in stems does not have a significant impact on N fixation. From the studies, there were no clear relationships between ARA and N concentration in varietal (Figures 1E,F) and sequential growth comparisons of Nipponbare (Figure 2). The high inorganic N conditions also suppressed N-fixing activity in free-living N fixers (Reed et al., 2011; Naher et al., 2018). Therefore, in this study, high inorganic N in the intercellular spaces was absent to inhibit N fixation, although further evidence is needed to support this conclusion.

*Oryza glaberrima*, such as CG14, which is collected from Casamance area in Senegal (Audebert and Fofana, 2009), are cultivated under low N input conditions (Sarla and Swamy, 2005). The CG14 was revealed to have the highest potential for BNF as CG14 showed the highest ARA and total sugar concentration (Figures 1A,B). It would therefore be interesting to examine whether the observed high N-fixing activity in *O. glaberrima* is related to the adaptability to low N conditions. Additionally, the two varieties of *O. glaberrima* (CG14 and WK18) accumulated significantly lower starch than the varieties of *O. sativa* (Table 2), which supported previous reports (Takasaki et al., 1994; Sakagami et al., 1999). Such lower starch and higher sugar concentrations in the stems of *O. glaberrima* are related to lower perenniality (Morishima et al., 1962) and higher stress tolerance of *O. glaberrima*, because *O. glaberrima* can grow in various harsh environments (Sarla and Swamy, 2005) where the large number of accumulated sugars is suggested to function in the stress responses. Furthermore, since this study used relatively limited varieties and genotypes, testing wider ranges of germplasms from diverse ecosystems would be useful to explore further promising rice genotypes.

### Dominant N-Fixing Bacterial Flora in Different Rice Endophytes

The four rice genotypes were separated from each other by the NMDS plot. This separation was based on the 16S-OTUs and NifH-OTUs compositions (Figures 4A,B). The results indicated that the bacterial community in the stem of each rice genotype would have both commonalities and specificity.

A common finding among all genotypes was that *Proteobacteria* was the most abundant phylum in both 16S rRNA and NifH analysis (Figures 5, 7). This finding was in agreement with the previous 16S rRNA analysis of the bacterial community in rice shoot and root (Ikeda et al., 2014; Edwards et al., 2015; Bertani et al., 2016), as well as in other plants, such as wheat (Gdanetz and Trail, 2017), *Panax notoginseng* (Dong et al., 2018), the root of *Trifoliumpratense* (Hartman et al., 2017), and *Brassica juncea* (Wang et al., 2020). A proportion of *Proteobacteria* was also reported to be significantly greater in rice roots than in the rhizosphere or bulk soil (Edwards et al., 2015), suggesting that *Proteobacteria* is a universally common phylum as endophytes.

Phylogenetic analysis of NifH obtained in this study also revealed that *Bradyrhizobium* sp. (*Alphaproteobacteria*) and *Paraburkholderia* sp. (*Betaproteobacteria*), both known as rhizobia, were the most dominant NifH-OTUs (Figure 8), which are considered to contribute the most to BNF in stems. Although these rhizobia were not dominant in the 16S rRNA analysis (Figure 6), a higher relative abundance of NifH analysis proposes that these rhizobia were dominant N-fixing bacteria. This result is similar to the report of active endophytic BNF by rhizobia, including *Bradyrhizobium* sp. and by non-rhizobial *Paraburkholderia* sp. in sugarcane stems, sweet potato stems, and tubers (Terakado-Tonooka et al., 2008; Fischer et al., 2012). In CG14, *Kosakonia* sp. was highly detected in addition to these rhizobia, which is proposed to have contributed to the highest N-fixing activity in the rice genotype.

So far, some members of the genus *Bradyrhizobium* and genus *Paraburkholderia* were isolated as rice endophytes or from rice paddy fields (Mano et al., 2007, Ishii et al., 2011; Piromyou et al., 2015; Hashidoko, 2018). *Bradyrhizobium elkanii*, a well-known soybean rhizobia (close to NifH-OTU I7), was reported as root-associated bacteria in rice (Mano et al., 2007). Also, *Bradyrhizobium* sp.TSA44 (close to NifH-OTU I6) was isolated from rice paddy soils in Japan (Ishii et al., 2011). Several *Bradyrhizobium* sp. have also been isolated as rice endophytes (Piromyou et al., 2015) and as plant growth-promoting bacteria of rice (Biswas et al., 2000; Padukkage et al., 2021). *Paraburkholderiamimosarum* (close to NifH-OTU I12–13), for example, was isolated from the rhizoplane of local rice in Indonesia (Hashidoko, 2018). Therefore, since *P. mimosarum* was originally isolated from the root nodules of *Mimosa* sp., that is grown mainly in wetland or seasonally flooded areas (James et al., 2001), the high abundance of *P. mimosarum* is proposed to be related to the waterlogged growth environment of the *Mimosa sp*. and rice. Some *Paraburkholderia* sp. have also been reported as rice endophytes, serving as plant growth-promoting bacteria of rice (Mattos et al., 2008; Coutinho et al., 2013).

Since most bacteria, close to the representative NifH-OTUs, have been reported to be capable of sugar (sucrose and/or glucose) utilization (Figure 8), the bacteria found in stems, found in this study, were possibly responsible for BNF by using those soluble sugars in rice stems although further confirmation by isolation of each strain is necessary. In particular, rhizobia, such as *Bradyrhizobium* sp. and *Paraburkholderia* sp. found in this study, would have degraded sucrose, glucose, and fructose. *Bradyrhizobium elkanii* USDA 76^†^ (95–97% identity with NifH-OTU I5–7), *Rhizobium jaguaris* CCGE 525^†^ (98% identity with NifH-OTU I3), and other bacteria belonging to the order *Rhizobiales* (*Rhizobium* sp. Ma-8, *B. diazoefficiens* USDA 110^†^, and *Bradyrhizobium* sp. MN-S) used sucrose, glucose, and fructose (Hameed et al., 2004; Dall'Agnol et al., 2013; Sajnaga and Jach, 2020) (Figures 6, 8). *Paraburkholderia mimosarum* PAS44^†^ (97–99% identity with NifH-OTUI10–13) can also metabolize glucose and fructose (Chen et al., 2006) (Figure 8). Since the translocated sucrose from leaves to stems is broken down into glucose and fructose by cell wall-bound invertase in the apoplast (Hirose et al., 2002), endophytes could also utilize those degraded monosaccharides (glucose and fructose) in the stem intercellular spaces. Additionally, many rhizobia use organic acids as a carbon source. Further investigation is needed to determine which carbon sources, including sugars, actually contribute to BNF in stem.

*Gammaproteobacteria* contributed to higher ARA of CG14 in addition to the rhizobia since bacteria belonging to *Gammaproteobacteria*, such as *Kosakonia* sp., were particularly high in the phylogenetic analysis of both 16S and NifH only in CG14 (Figures 6, 8). *Kosakonia oryzae* Ola 51^†^ (100% identity with NifH-OTUI24), isolated from roots in wild rice, can fix N at low pO_2_ (Meng et al., 2015) and use many kinds of sugars, including sucrose, glucose, and fructose (Qiu et al., 2017). The high concentration of sugars also become microbially accessible carbon sources in CG14, thereby leading to a hypoxic environment as aerobic bacteria consume oxygen, which becomes a favorable environment for *Kosakonia* sp. Another possibility of the higher abundance of *Kosakonia* sp. than *Bradyrhizobium* sp. in CG14 is because *Bradyrhizobium* is an oligotrophic bacterium (Saito et al., 1998; Okubo et al., 2012). It would therefore be interesting to further examine whether the dominance of *Gammaproteobacteria* is related to higher ARA in CG14 with high sugar accumulation.

The low abundance of methane-oxidizing bacteria, such as *Methylocella tundrae* T4^†^ (close to NifH-OTU I8) and *Methylocystis parvus* OBBP^†^ (close to NifH-OTU I9), in NifH analysis indicated that their contribution is minor in the stem, unlike the roots, which is explained by the lower abundance of methane in the stem than the root. In rice roots, Bao et al. (2014) reported that the Nif peptide was most abundantly expressed by *Methylosinus, Methylocella*, and *Methylocystis*. They suggested that significant contributions in N fixation are associated with methane oxidization.

As mentioned above, aerobic N fixation is active in rice stems since most of the representative OTUs, including *Bradyrhizobium* sp., *Paraburkholderia* sp., and *Kosakonia* sp., were classified as Cluster I in NifH (Figure 8), which contains primarily aerobic and facultatively anaerobic bacteria (Gaby and Buckley, 2014). Although NifH-OTUs are classified as Cluster III, which are exclusively in obligate anaerobes (Gaby and Buckley, 2014), they were relatively low in abundance (Figure 8). Similarly, absolute anaerobic bacteria in stem BNF are proposed to have collaborated with aerobic bacteria to some extent. Co-cultures of N fixer and aerobic heterotrophs were therefore reported to enhance N-fixing activities (Okuda and Kobayashi, 1961). Also, a bacterial consortium, consisting of both aerobic and anaerobic bacteria, was suggested to create an anaerobic microsite that favors anaerobic N fixation in the plant (Minamisawa et al., 2004). In Cluster II, several NifH-OTUs closely related to *Brooklawnia* sp., a facultatively anaerobic *Actinobacteria* (Bae et al., 2006), has also been found in higher abundance with low identity; however, the sequences matched or closely matched that of uncultured bacterium collected from rice plants in Japan (Figure 8, Supplementary Table 3). These results indicate that these bacteria are universally present in rice plants, although their function remains unknown. Besides, the contribution of endophytes belonging to Cluster II with alternative nitrogenase sequences would be small. Alternative nitrogenaseis, a paralog of NifH, contains an Fe-Fe cofactor in place of the Fe-Mo cofactor found in NifH (Masepohl et al., 2002). Alternative nitrogenase functions particularly under Mo-deficient conditions (Bishop et al., 1982), but less efficiently in N_2_ reductions compared to Nif (Eady, 1996). Since rice plants grown in 2018 did not show any Mo deficiency symptoms, BNF by alternative nitrogenase has also made a minor contribution to this study.

**Figure 9:**
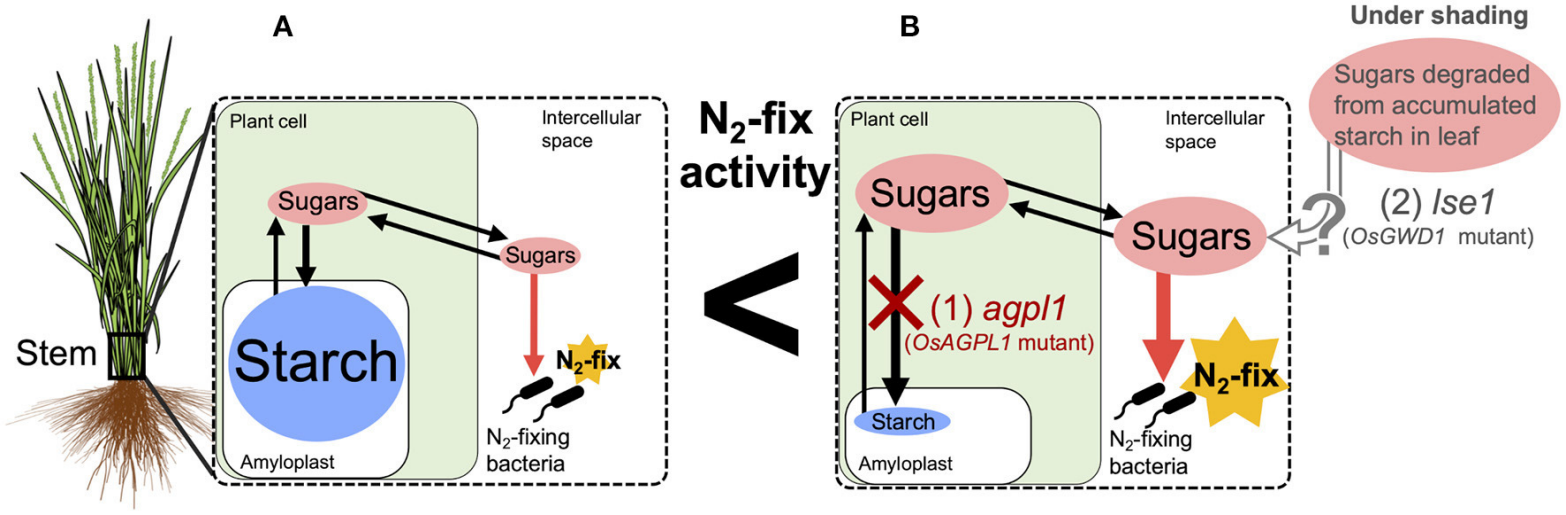
Hypothetical model of the relationship between nitrogen (N) fixation and sugars in the stem of rice. Endophytic N_2_-fixing bacteria are expected to use sugars (sucrose and/or glucose) in the intercellular spaces as carbon (C) sources for N fixation. **(A)** Most *Oryza sativa* varieties, such as Nipponbare, accumulate starch as a major non-structural carbohydrate in their stems with small amounts of sugar in the intercellular space, which may be utilized by N_2_-fixing bacteria. **(B)** CG14, a variety of *O. glaberrima*, and *agpl1*, a mutant lacking a starch synthesis gene in shoot (1), accumulate sugars in the parenchyma. *lse1*, which lacks a starch degradation gene in leaf and accumulates high starch in leaves (2), is supposed to translocate sugars derived from starch to the stem under the shading treatment. Higher amount of sugars in the intercellular spaces possibly enhance N fixation.

In summary, this study revealed that the N-fixing activity in rice stems was positively affected by accumulated soluble sugars (Figure 9). This implies the possibility of further genetic improvements of rice by manipulating sugar accumulation. The N-fixing endophytes, known as rhizobia, may fix N while utilizing the sugars based on NifH analysis. For ARA and microflora analysis, we used bulk samples from the 10 cm lower part of the stem of which the upper parts are under aerobic conditions above the water surface and the lower parts are under semi-aerobic to anaerobic conditions in water and soil. Therefore, further examination should clarify the relative importance of aerobic N-fixing and anaerobic N-fixing bacteria, such as Cluster III and methane-oxidizing bacteria, as well as their joint N fixation in different locations. Precise analysis of the bacterial flora in the apoplast is required since it cannot be denied that the bacteria detected in this study partly included those attached to the surface of stems. Furthermore, physiological and quantitative evaluation of sugar usage in these stems is required, in relation to the N fixation by each microflora to elucidate their contribution to N nutrition in rice.

## Data Availability Statement

The datasets presented in this study can be found in online repositories. The names of the repository/repositories and accession number(s) can be found in the article/Supplementary Material.

## Author Contributions

TO, RS, and MK conceived the study and designed the experiments. TO and RS performed the overall experiments. AT and DS contributed in amplicon analysis and plant analysis, respectively. TO, RS, AN, and KU contributed in data analysis. AN contributed in phylogenetic analysis. TO synthesized the data and wrote the manuscript. RS, AN, KU, AT, DS, and MK contributed in reviewing and editing. All authors contributed to the article and approved the submitted version.

## Conflict of Interest

The authors declare that the research was conducted in the absence of any commercial or financial relationships that could be construed as a potential conflict of interest.

## Publisher's Note

All claims expressed in this article are solely those of the authors and do not necessarily represent those of their affiliated organizations, or those of the publisher, the editors and the reviewers. Any product that may be evaluated in this article, or claim that may be made by its manufacturer, is not guaranteed or endorsed by the publisher.
